# Moderating Effect of Dynamic Environment in the Relationship between Guanxi, Trust, and Repurchase Intention of Agricultural Materials

**DOI:** 10.3390/ijerph16193773

**Published:** 2019-10-08

**Authors:** Lan Li, Gang Li, Xiaoling Feng, Zhigao Liu, Fu-Sheng Tsai

**Affiliations:** 1 Research Center of Henan Economy, School of Business Administration, Henan University of Economics and Law, Zhengzhou 450046, China; lilan@huel.edu.cn; 2 School of Management and Economics, North China University of Water Resources and Electric Power, Zhengzhou 450045, China; 201510751237@stu.ncwu.edu.cn; 3 Jiangsu FoxLink Green Energy Technology Co. LTD., Lianyungang 222000, China; bob_liu@foxlinkpower.com; 4 Department of Business Administration, Cheng Shiu University, Kaohsiung 83347, Taiwan; 5 Center for Environmental Toxin and Emerging-Contaminant Research, Cheng Shiu University, Kaohsiung 83347, Taiwan; 6 Super Micro Mass Research and Technology Center, Cheng Shiu University, Kaohsiung 83347, Taiwan

**Keywords:** guanxi, trust, dynamic environment, repeated purchase intention, agricultural food system

## Abstract

Repurchasing intention of agricultural materials is a key to a sustainable food business system. The novel contribution of this study is that we go beyond technical aspect and look into human capital dynamics in a general context, by examining how different dimensions of ‘guanxi’ (i.e., personal relations and instrumentality) between farmers and agricultural retailers affect trust between the two and, in turn, repeated purchase intention of agricultural materials by farmers in China. To further generate implications for food system as a whole, we also examined how dynamic environment moderates the effects mentioned above. Adopting survey method and multivariate analyses, this study tests the hypotheses with a collected data set of 578 farmers from representative rural areas of China. The results show that guanxi between farmers and agricultural retailers has a positive effect on trust between them and on repeated purchase intentions of farmers. While instrumentality has a negative effect on trust between them and on repeated purchase intentions of farmers. The trust between farmers and agricultural retailers promotes farmers’ repeated purchase intentions. The intensity of competition negatively moderates the positive relation between trust and repeated purchases. Demand uncertainty does not moderate the positive effect of trust on repeated purchases. The results and discussion shed light on agricultural food system sustainability from a dynamic environment embedded business relationship perspective.

## 1. Introduction

The competition in the agricultural material market of China is becoming more intense in recent years as the market economy in this sector is developing very fast. A saying that is popular among marketing practitioners is that “the cost of attracting new customers is five times that of maintaining old ones”. Agricultural retailers must focus on cultivating farmers’ repeated purchase intentions to generate satisfactory sales. Thus, exploring the factors that stimulate farmers’ repeated purchase intentions is of great importance for agricultural retailers, and this question also deserves more research attention from academia.

Previous studies on customer repeated purchase intentions show that factors such as perceived value [1,2], customer satisfaction [3,4], customer trust [5,6], and guanxi [7,8,9] are important antecedents. As the business culture and environment is very unique in China, a group of scholars have explored the catalysts of repeated purchase intention in China. They found that the guanxi has positively promoted customer purchase intention [8,9]. Trust plays an important role in customer repeat purchases by reducing information asymmetry, and customer perceived risk, thus forming a positive intention of purchasing [10,11]. 

In a business-to-business (B2B) context, trust is also important in establishing a stable, continuous, and good supplier relationship, thereby enhancing the role of corporate customer share and customer retention [12]. Moreover, studies also find that, unlike the trust established in the legal provisions and written contracts in Western society [13], guanxi in Chinese society are the foundation of trust establishment [14,15]. It facilitates the establishment and development of interpersonal trust between enterprises [16].

However, this research on guanxi, trust, and repeated purchase intentions is mostly based on B2B or online marketing scenarios. The purchase of agricultural materials in rural area of China is business-to-customer (B2C) and mostly offline transactions. In addition, the agricultural materials market in China is very unique. Most farmers in China have very limited knowledge of agricultural materials such as pesticides, seeds, and fertilizers. They do not know the components or usage of them [17]. Therefore, farmers are heavily relied on the retailers’ recommendation, and their purchasing behavior is restrained within the network with retailers [18]. In addition, fake pesticides, seeds, and fertilizers have made farmers more dependent on retailers who they have built trust with so as to get a sense of security. Thus, the importance of guanxi and trust is even more significant in the agricultural material market of China. The conclusions drawn by the extant research in the context of B2B and online marketing are unlikely to be able to explain the agricultural materials transactions in China. An independent investigation on the antecedents of repeated purchase intention of agricultural materials by farmers in China is necessary. 

Different from western countries, research shows that the relationship between the business parties in China is of great importance in business transactions. It is a stepping stone of making a deal successfully. The level of trust between the buyers and sellers determines the way in which business is done [14]. More specifically, customer purchasing in China begins with guanxi, and trust is about whether the two parties can cooperate for a long time. This situation makes guanxi and trust a natural focus of research on trading relationships in China, especially in the rural area where the Chinese traditional culture of relationship-based society is dominant. 

In addition, given the importance of environment in determining business activities, it is necessary to ask the question that will the relationship between guanxi, trust, and repeated purchase intentions be affected by the boundary conditions, and ‘what are the implications for the food system?’. Besides being embedded deeply with Chinese traditional culture of relationship-based “acquaintance” society, the agricultural materials market in China is emerging and developing. The number of agricultural retailers is growing rapidly, and farmers increasingly enjoy more choices. In a dynamic environment such as this, can trust, as a marketing tool, still significantly increase repeat purchase intentions? This is also a question that needs to be addressed.

In view of the above questions, based on relationship marketing and social exchange theory, from a perspective of Chinese ‘relationships’, this paper investigates firstly, the impact of the two dimensions of guanxi (i.e., personal relations and instrumentality) on trust. An emotional relationship is a relationship based on human emotions and established by a person to satisfy the emotional needs of both parties [15]. The instrumental relationship refers to the social relationship established by individuals with others in order to achieve some goals [15]. Secondly, the effect of trust on repeated purchase intentions. Thirdly, the moderating effects of demand uncertainty and competition intensity in the dynamic environment on the relationship between trust and repeated purchase intentions. 

The following section will review the relevant literature and theories, develop the conceptual framework, and propose the hypotheses. The research methods and results are given in the third section. The conclusions, limitation of the research, and the direction of further studies are discussed in the last section.

## 2. Theoretical Base and Hypotheses

### 2.1. Theoretical Base

#### 2.1.1. Guanxi

‘Guanxi’ in China refers to the interrelationship between people that is based on personal emotions and interests, and established in the process of social activities in the context of Chinese culture [8]. China is a country with a relationship-based culture. People are concerned about the establishment, maintenance, and expansion of relationships. Guanxi is considered very important in everyday life, as well as economic and political activities [16]. Unlike the self-independence consciousness promoted by the West, China’s guanxi shows a “difference sequence pattern” structure that is “self-centered” [17]. That is to say, Chinese culture emphasizes that individuals need to know where they are in the network of guanxi and should have different strategies for dealing with different people [18].

Guanxi, as a culturally embedded variable, is complex and contains multiple dimensions [19]. Some studies equal guanxi to personal relations, and test its impact on speculation [20,21]. Others categorized cross organizational guanxi into emotional and instrumental groups [22,23]. Given the purpose of relationship and communication, a few studies have categorized the guanxi in China into an emotional relationship, an instrumental relationship, and a mixed relationship [15]. In a study of inter-enterprise transaction purchase behavior, guanxi was divided into four dimensions, i.e., personal relations, human condition, face, and instrumentality [8].

A combination of the traditional ‘relationship-based’ and a modernized ‘commercial economy’ type of purchasing behavior is spearing in the rural area in China in recent years. The purchase behavior of farmers is increasingly instrumental. The purchase decision of agricultural materials is no longer simply based on the emotional relationship of blood, kinship, or geography, but increasingly reflects the instrumental orientation of pursuing economic and utilitarian ends [24]. It is this interweaving of the traditional pattern of difference sequence and the modern commercial economy that leads to the complexity of the formation of purchase decision of farmers and reflects a mixed guanxi. Therefore, this paper divides the guanxi between farmers and agricultural retailers into emotional and instrumental relationships. 

An emotional relationship is a relationship based on human emotions and established by a person to satisfy the emotional needs of both parties [15]. In the ‘acquaintance’ society in rural areas of China, the relatively closed and homogeneous guanxi network means that the emotional relationship between people is mostly based on kinship or geographical relationship. While guanxi refers to blood, kinship, or ‘symbolic’ kinship including fellow villagers, friends, classmates, etc. [25]. Therefore, in most cases, emotional relationship equals to personal relations. In agricultural marketing, farmers’ trust in agricultural retailers is based on frequent interactions between the two parties. It is a kind of interpersonal trust, reflecting farmers’ confidence in the reliability and integrity of agricultural retailers.

The instrumental relationship refers to the social relationship established by individuals with others in order to achieve some goals [15]. In the instrumental relationship, the orientation of people’s communication is very obvious, that is, the relationship is a means to achieve the goal. The instrumental relationship is different from the personal relationship. Its emotional component is negligible. The parties to the transaction have always adhered to the fairness principle to ensure their own interests are protected. The long-term orientation of the relationship is usually very low. Yang examined the interpersonal relationship to the market economy and proposed that the contractualization of interpersonal relationship would replace the emotionalization [26]. Liu also believes that the interpersonal relationship under the conditions of market economy is generally weakening in emotional components and strengthening in economic interests [27]. In agricultural materials transactions, farmers want to establish good interpersonal relationships with agricultural retailers in order to purchase pesticides, fertilizers, and seeds with low costs and better benefits. Instrumental relationships are a means to achieve a goal, so they are short-lived and unstable [8], which is not conducive to the enhancement of customer trust and the promotion of repeated purchase intentions.

#### 2.1.2. Trust

Trust refers to the confidence of one party in the transaction relationship to the reliability and integrity of the other party [5]. Trust is an important part of relationship marketing theory. It is a key variable that determines the success or failure of relationship marketing and can promote the establishment of a complete partner-type trading relationship. In Chinese rural areas, the production and living space of farmers is relatively limited, the social network has a high degree of closeness and homogeneity, which makes the role of trust more indivisible [28]. In addition, farmers often lack sufficient professional knowledge of agricultural materials, thus agricultural retailers are often their main source of information. Therefore, farmers rely heavily on the trust of agricultural retailers. Furthermore, farmers face a high risk of buying fake and shoddy agricultural materials, and thus are more inclined to choose an agricultural retailer whom they trust. Therefore, in order to purchase high-quality agricultural materials with reasonable price, it is prominent for farmers to establish trustworthy relationship with retailers. Trust sets up a bridge between farmers and agricultural retailers, which improves the efficiency of interaction, reduces unnecessary conflicts, and increases the possibility of cooperation, and promotes repeated purchase intentions.

#### 2.1.3. Repeat Purchase Intention

The influencing factors of repeated purchase intentions have been a hot topic in the research of marketing. In the mid-1980s, customer perceived value was considered to be the most important factor affecting customers’ repeated purchase intentions, and it was also one of the important methods to measure whether enterprises have competitive advantage in service [1,2]. Customer perceived value has a direct impact on the way in which repeated purchase intentions work, so as indirect effects through customer satisfaction. In the early 1990s, relationship marketing theory attracted more attention. Scholars used this theory as a starting point to argue that customer satisfaction is a major driving factor for customers’ repeated purchases [3,4]. 

In the mid-to-late 1990s, customer trust stood out among the influencing factors of repeated purchase intentions and was highly valued by research scholars. It believed that trust had a significant effect on the generation of repeated purchase intentions [5,6]. Since the beginning of this century, guanxi has become the focus of attention of the research on factors affecting repeated purchase intentions [7,9]. Unfortunately, this research is either conducted in Western countries or based on B2B or online marketing scenarios [7,11,14]. 

While agricultural marketing has its uniqueness compared to B2B or online marketing. Firstly, agricultural materials are distinguished from consumer products and living materials by their high aftereffect—i.e., the cost of purchasing fake and shoddy products is very high—and the effect of the use of agricultural materials can only be identified in the middle, late, or end of the agricultural production process. Secondly, the main body of purchase of agricultural materials in China is thousands of individual farmers with small farms. The expertise to purchase agricultural materials is poor. Most of them make the purchase according to previous farming and purchasing experience, and are easily affected by the surrounding voices. 

Thirdly, different from other trading networks, farmers are at the edge of the rural social network; their ability to effectively obtain information and resources is weak. Agricultural retailers often deal with supply and marketing companies, and have many channels to obtain information. Therefore, they occupy a central position in the agricultural social network, and have control over information and resources. Thus, farmers depend heavily on agricultural retailers for information and suggestions [17]. Therefore, whether the relevant conclusions that are drawn from B2B or online marketing scenarios are applicable in the context of agricultural materials needs further verification.

Social exchange theory believes that interpersonal interaction is a process that both parties follow the principle of reciprocity [29,30]. The value and benefits obtained from the interaction directly promotes the individual’s willingness to identify, attach, and maintain relationships with each other. In order to continue to benefit from the interaction between the two parties, the individual is motivated to perform actions beneficial to the other party, so as to construct and maintain relationship. The establishment of the farmer–agricultural retailer relationship means that the agricultural retailers provide the resources and services that meet the farmers’ needs in exchange for the farmers’ repeated purchase intentions. Farmers’ repeated purchases make the agricultural retailers achieve better sales performance. This behavior falls into the category of social exchange.

In agricultural trading, repeated purchase intentions reflect the desire and inclination of farmers to maintain a relationship with a certain agricultural retailer. Due to the cyclical nature of agricultural production and the characteristics of dedicated asset investment, farmers will have a strong motivation in cooperation with agricultural retailers to ensure a desirable result of farming. The relatively closed and acquaintance-driven society gives guanxi a key position in influencing the repeated purchase intention of farmers. The trust in agricultural retailers is the guarantee for farmers to deal with the purchase risk. Guanxi positively influences farmers’ repeated purchase intention through establishing trust between agricultural retailers and farmers. 

#### 2.1.4. Dynamic Environment

A dynamic environment is the most prominent feature of an enterprise’s external environment. The economic behavior of both seller and purchaser is always embedded in a certain social environment, and is bound to be affected by environmental factors [17]. A dynamic environment makes agricultural retailers get into a state of dynamic change and uncertainty, which will have a major impact on their marketing decisions.

Farmers and other retailers are the two most important market players in the agricultural market for agricultural retailers. The rapid and ever-changing demand of farmers has put pressure on agricultural retailers. The huge group of competitors and the fierce competition in the industry has clearly formed a big obstacle for the retailers to achieve competitive advantages and business expansion. Therefore, this study considers that demand and competition are the two most important aspects of environmental factors that influence the repeated purchase intentions of farmers [31,32]. Demand uncertainty refers to the extent that customers seek new and different products and services, changes in the demand, and the difficulty of predicting of the changes by retailers. Competitive intensity refers to the degree of similarity, renewal speed, competitive strategy, and competitive incentives of the services or products offered by competitors [31,32,33].

The demand of agricultural materials is vulnerable to fluctuations in market conditions during the sales process. Therefore, compared with general consumer goods and industrial products, agricultural product marketing faces higher demand uncertainty and changes in competition intensity [21]. The dynamic environment will cause uncertainty in the transactions between farmers and agricultural retailers, which in turn will affect the cooperative relationship between the two parties. In an environment with high demand uncertainty and strong competition intensity, the operation of agricultural retailers faces greater risks and pressures and farmers will also be indecisive in trusting in agricultural retailers in this environment. Therefore, this paper further explores the moderation effect of demand uncertainty and competition intensity on the relationship between trust and the repeated purchase intention of farmers. 

**Figure 1:**
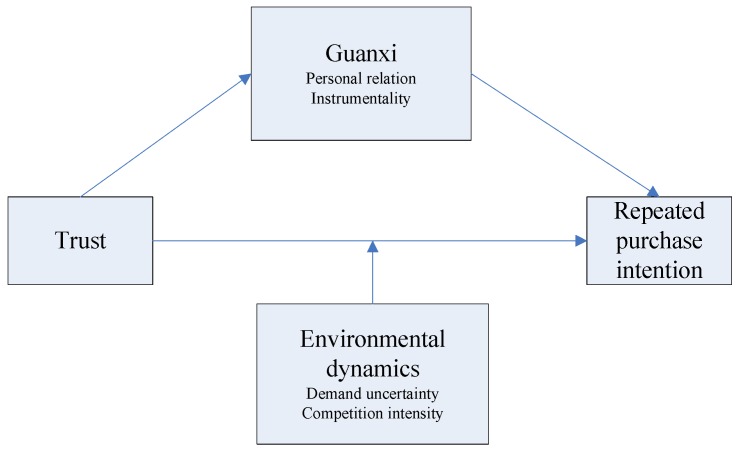
Conceptual model.

In summary, we propose a conceptual model in which guanxi between farmers and agricultural retailers improves the trust between them and the repeated purchase intentions of farmers, trust promotes the repeated purchase intention of farmers, and this effect is moderated by environmental dynamics. The conceptual framework is depicted in Figure 1.

### 2.2. Research Hypothesis

#### 2.2.1. The Impact of Guanxi on Trust

• Guanxi and Trust

The close guanxi between farmers and retailers indicates that the relationship has been tested for a long time and the two sides believe that the other party is a friend of their own. Agricultural retailers will not forge the information on quality and other aspects of agricultural materials. Instead, they will really care about the agricultural harvest and benefits of farmers and provide quality products and services that meet the needs of farmers. This will increase farmers’ confidence of agricultural retailers in fulfilling their responsibilities and maintaining credibility. Therefore, farmers believe that agricultural retailers with good guanxi are worthy of trust and have the ability to recommend suitable products for them. In addition, in the acquaintance society of Chinese rural area, the close guanxi indicating that the two parties will not only be friendly and mutually supportive at work, but also extend the friendship to non-work issues, and the two sides will maintain contact. Frequent communication and interaction play a role of mutual benefit and eliminates contradictions. Thus, farmers have higher trust in agricultural retailers. As Huang (2015) argued that in the context of micro-marketing, guanxi can promote the generation of trust [18]. Therefore, it is proposed that all else being equal,

**Hypothesis** **1.** *The closer the personal relations (i.e., higher guanxi) between farmers and agricultural retailers are, the higher the trust between them.*

• Instrumentality and Trust

On the contrary, if farmers only contact agricultural retailers when they want to buy agricultural materials, the relationship is established by the farmers solely to meet their goals, to achieve possible benefits, to do things smoothly and obtain internal information. In other words, both the farmers and the agricultural retailers treat the relationship as an instrumentality and will not trust each other. 

Based on this short-term trading relationship, it is difficult for agricultural retailers to truly care about the agricultural harvest and interests of farmers. Naturally, the reliability of agricultural retailers perceived by farmers will be reduced. In addition, the trust established within the instrumental relationship is short-lived and unstable, or misplaced. Once other agricultural retailers can offer more favorable prices, more considerate services or more adequate agricultural information, the trust relationship is likely to collapse. Therefore, it is proposed that, all else being equal,

**Hypothesis** **2.** *The more instrumental the relationship between farmers and agricultural retailers is, the less possible the trust between them will be established.*

#### 2.2.2. Impact of Guanxi on Repeated Purchase Intentions

• Personal Relations and Repeated Purchase Intention

The emotional elements of guanxi established a foundation of cooperation between farmers and agricultural retailers. The better the personal relations, the more likely they form an ‘inside circle’. In this circumstance, even if they do not consider doing business with the other side, the two parties will care about each other’s private affairs and help each other, showing the responsibility and support between the members of the ‘inside circle’. This kind of responsibility and affection not only weakens the motivation of agricultural retailers to pursue profit at the expense of morality, but also enables farmers to generate more business contacts with agricultural retailers in the future, which in turn will stabilize the personal relations between the two. The long-term stable relationship plays an important role in retaining customers and improving customers’ repeated purchase intentions. In addition, better personal relations also mean that farmers will get more price concessions, credit sales services, and agricultural materials information in the actual purchase process. Thus, farmers are willing to buy again because of the perception of ‘returning the favor’. Therefore, it is proposed that all else being equal,

**Hypothesis** **3.** *The better the personal relations between farmers and agricultural retailers, the stronger the intention to repeat purchases.*

• Instrumentality and Repeated Purchase Intention

In the rural society of China, the role of instrumental rationality is apparent in the purchase decision of farmers choosing agricultural retailers [34]. The continuous deepening of reform and opening of the market has enabled more agricultural materials to enter the business. Farmers have more autonomy and initiative in the choice of agricultural materials, which makes the purchase of agricultural materials more utilitarian. Some farmers only contact agricultural retailers for buying agricultural materials, and this instrumental relationship is purely of interest. The more a farmer thinks that the relationship between him and the agricultural retailer is based on the instrumentality, the more likely it is that he will only consider which agricultural retailer can provide the maximum benefit the next time of the agricultural purchase, and tlower repeated purchase intentions is guaranteed. Therefore, it is proposed that all else being equal,

**Hypothesis** **4.** *The more instrumental that farmers are in their relationship with agricultural retailers, the lower their intention to repeat purchases.*

#### 2.2.3. Impact of Trust on Repeated Purchase Intentions

Individual consumers purchasing decisions are closely related to their trust in the seller [18]. The trust of farmers in agricultural retailers means, firstly, farmers believe that agricultural retailers will keep their promises during the transaction, guarantee the quality of agricultural materials, and provide services at preferential prices and with satisfactory technology, i.e., no false information about the quality, price, or service of agricultural materials will be provided to induce farmers’ purchase. Secondly, farmers believe that agricultural retailers are always concerned about their interests, and really care about the agricultural harvest of farmers. Thirdly, farmers’ ability to freely obtain effective information is limited, and the asymmetry of information makes farmers always in a weak position in the process of business transactions. In this case, trust acts as a compensation mechanism to effectively avoid the speculation of agricultural retailers. Farmers believe that agricultural retailers are trustworthy, because these retailers also believe that doing so will increase farmers’ intentions of repeat purchases and maintain long-term and stable trading relationships benefit both sides. Therefore, it is proposed that all else being equal,

**Hypothesis** **5.** *Farmers’ trust in agricultural retailers positively affects farmers’ repeated purchase intentions.*

#### 2.2.4. Moderation Role of Dynamic Environment Played in the Relationship between Trust and Repeated Purchase Intentions

• Moderation Effect of Demand Uncertainty

In an environment with high demand uncertainty, farmers’ trust in agricultural retailers will be more effective in promoting repurchase intention. In the condition of high demand uncertainty, farmers’ demand preferences for agricultural materials appear inconstant and always seek something new and different, which indicates some risks in transferring from one brand or type to another brand or type of fertilizer, pesticides, and seeds. Therefore, farmers will choose a trusted agricultural retailer to avoid potential risks and provide protection for their replacement options. Firstly, farmers believe that the agricultural retailers they trust will guarantee the quality of newly purchased agricultural materials and provide corresponding technical services. Secondly, they believe that retailers will not exaggerate the quality of new agricultural materials to induce farmers to buy. Thirdly, farmers believe that retailers will really care about the agricultural harvest after farmers use new agricultural materials. This will further enhance farmers’ intention to repeatedly purchase agricultural materials. Therefore, it is proposed that all else being equal,

**Hypothesis** **6.** *Demand uncertainty positively moderates the relationship between trust and repeated purchase intentions.*

• The Moderation of Competition Intensity

A highly competitive environment reduces the incentives for trust to promote repeat purchase intentions. On the one hand, the increase in competition intensity indicates that many agricultural retail stores are in the market which providing similar agricultural materials, and the change of agricultural materials is very fast. In this situation, in order to be competitive, agricultural retailers will try their best to improve service, e.g., keep their promises in agricultural materials transactions, provide preferential prices and professional technical services, and go deep into the fields to actively support farmers of the use of agricultural materials and actual harvest. Farmers will thus not worry about the provision of false information or inferior agricultural materials by a certain agricultural retailer, and they can compare and replace agricultural retailers more easily. On the other hand, in order to cope with fierce competition, agricultural retailers will adopt strategies such as low-cost concessions, home delivery, and technical services. These concessions and convenience services will attract farmers to switch from formerly trusted agricultural retailers to new agricultural retailers. Therefore, it is proposed that all else being equal,

**Hypothesis** **7.** *The intensity of competition will negatively moderate the relationship between trust and the repeated purchase intention of agricultural materials.*

## 3. Materials and Methods

### 3.1. Sampling and Data

A questionnaire approach is adopted by the study to collect data. The questionnaire collectors are mainly university students who were born and grow up in rural areas of China, so that they were familiar with the production and life in rural areas. They were trained on the skills required for the survey before going to do the data collection. In order to ensure the recovery rate and efficiency of the questionnaire, from July to September 2016, a stratified sampling method was adopted to select the samples. Firstly, a province with developed agriculture was randomly selected from the provinces and autonomous regions in the eastern, central, and western regions of China. Hebei province was drawn from the eastern region; the central region was drawn to Henan Province, and the western region was drawn to Guizhou Province. Because agriculture is a pillar industry in the western region, in order to make the research questions of this paper more thorough, Ningxia Province was selected from the west. The agricultural development conditions of the four provinces are as follows: Most of Hebei Province belongs to the North China, with more arable land and better sunshine. It is one of the national grain and cotton oil production areas and one of the country’s 13 commodity grain production bases. Located in the middle and lower reaches of the Yellow River in central China, Henan Province has a humid climate, abundant rainfall and long sunshine hours. It is an important agricultural production base and agricultural province in China. The climate of Guizhou Province is mild, humid, rich in heat, and the conditions for agricultural development are good. Ningxia belongs to the plain area, with long sunshine hours, high effective accumulated temperature, sufficient precipitation and long frost-free period, and the agricultural foundation is unique.

Secondly, from the four provinces of Henan, Hebei, Ningxia and Guizhou, 10 cities with relatively developed agriculture—such as Luohe City, Baoding City, Yinchuan City, and Zunyi City—were randomly selected. Among them, Luohe City is located in Henan Province. In the south, warm and humid monsoon climate, four distinct seasons, more precipitation, better agricultural development foundation; Baoding City has four distinct seasons, sufficient sunshine, heat, and rain in the same season; Yinchuan City is located in Hetao Plain, an important irrigated agricultural area. Zunyi City has a large cultivated land and distinctive industries. Thirdly, 13 counties were selected with strong agricultural development advantages such as Linyi County, Tang County, Xixia District, and Wuchuan County from these 10 cities. Finally, in these counties, select some representative villages and farmers were selected in these counties. 

Door-to-door visits were used to issue and collect questionnaires. The respondents selected are the farmers who are mainly engaged in agricultural production in each family and have experience in purchasing agricultural materials. A total of 605 households were selected for the survey, and 605 questionnaires were collected. After eliminating 27 invalid questionnaires, the effective questionnaire was 578. The effective recovery rate is 95.5%. The main reason for the invalidity is that it is not completed and/or there are extensive default values in the purification process of the questionnaire. A profile of the samples is shown in Table 1.

A pilot study was firstly conducted in a typical agricultural village in Henan Province in June 2016. 80 questionnaires were collected, of which 75 were valid, and the effective recovery rate is 93.75%. According to the information feedback of the interviewees and the data analysis results of the 75 answers of the pilot study, the questionnaire was further revised, and a formal questionnaire was finally formulated. To ensure the authenticity and validity of the survey data, all data collected in the pilot survey is not used as a final sample.

Harman’s single factor test was conducted to ensure that common methods variance is not a threat to the study. All items in the questionnaire were put together for un-rotated factor analysis and a total of six principal component factors were extracted. The first factor explains 25.2% of the total variance, much lower than the threshold value of 40%, which means that the common method variance is not significant and will not affect the conclusion of the study.

### 3.2. Measurement 

The measurement of the variables is based on extant literature [8,16,31,32,35], and adjusted and optimized according to the pilot study. For indicator purification, the items with the lower values of the factor loadings or the corrected item total correlation (CITC) is deleted by SPSS (version 22, IBM, New York, NY, USA), and were conceptually confirmed by all co-authors. Generally, if CITC is less than 0.4, the item is suggested to be deleted, because this means this item could not be well combined with other items to reflect the measured construct (variable) [36]. Guanxi is measured based on Wang et al. [16] and Li [8]. It is divided into two dimensions of personal relations and instrumentality, in total seven items. Trust mainly draws on the scales of Doney and Cannon et al. [35], a total of six items. Repeated purchase intention is measured by the scales developed by Paolo and Laurent [37], a total of three items. The dynamic environment includes two dimensions of demand uncertainty and competition intensity. Based on the scales developed by Yang et al. [31] and Jaworski and Kohli [32], they are measured by three items respectively. Table 2 shows the details. See Appendix A for the full questionnaire. 

### 3.3. Reliability and Validity 

To ensure the reliability and accuracy of the measurement results, this study firstly tested the reliability and validity of the data. Using internal consistency as a measure of reliability, Table 1 shows that the Cronbach’s α of each scale reached 0.6 or more. In addition, the composite reliability values (CR) of each variable are greater than 0.7, and even close to or higher than the 0.8, which indicates that the internal consistency of the data is acceptable, and the variables of the questionnaire have good reliability.

As shown in Table 3, the Kaiser-Meyer-Olkin (KMO) values of each variable are greater than 0.5, and the Bartlett sphericity test has a significant probability of 0.000, indicating that each variable is suitable for factor analysis. The results of the factor analysis are shown in Table 1. The factor loadings of each variable are greater than 0.6, which is much higher than the standard of 0.4. The average variance extraction value (AVE) of all variables exceeds 0.5, indicating that the scale has good convergence validity. In addition, as shown in Table 3 below, the AVE square root of each variable is greater than the correlation coefficient of other variables, which meets the requirements of the discriminant validity test, indicating that the questionnaire has good validity. Furthermore, the percentage of variance explained of the factors is greater than 50%. It can be seen that the designed metrics have a higher degree of interpretation of the research variables and can truly measure the research variables. In summary, the scales used in this paper have good reliability and validity.

Except for the AVE of all variables, the mean, standard deviation, and correlation coefficient of each variable are shown in Table 4. 

## 4. Results

### 4.1. Results of Regression Equation Modeling

In order to ensure the accuracy of the empirical results, the variables are sequentially placed into the regression equation model by the hierarchical regression method, and the influence of the predicted independent variables on the dependent variables is analyzed by comparing the changes of the regression coefficients. The results are shown in Table 5. In order to minimize the collinearity problem between interaction variables, the related variables were averaged before regression analysis. The independent and moderation variables are centralized and then multiply for the moderation analysis. 

For the influence of personal relations and instrumentality on trust, model 1 of Table 4 shows that the model fits the data well (R^2^ = 0.190). In the regression results, the personal relations are significantly positively related with trust (β = 0.437, *p* < 0.01), thus Hypothesis 1 is supported. Instrumentality is significantly negatively related with trust (β = −0.063, *p* < 0.1), thus Hypothesis 2 is supported. For the effect of personal relations and instrumentality on repeated purchase intention, it is known from model 2 that the fit is better (R^2^ = 0.343). In the regression results, the personal relations are significantly and positively related with the repeated purchase intention (β = 0.215, *p* < 0.01), thus the Hypothesis 3 is supported. The instrumentality is significantly and negatively related with the repeated purchase intention (β = −0.148, *p* < 0.01), thus Hypothesis 4 is supported. The effect of trust on repeated purchase intentions is also shown in model 2. In the regression results, trust is significantly and positively related with the repeated purchase intention (β = 0.443, *p* < 0.01), thus Hypothesis 5 is supported.

The three-step test of hierarchical moderating regression method is adopted to examine the moderation effect. The detailed approach is as follows. Firstly, the impact of trust on the intention of repeated purchase is tested. It can be seen from model 2 that trust has a significant positive impact on the repeated purchase intention. Secondly, the influence of trust and demand uncertainty on the repeated purchase intention is examined. The model 3 fits the data well (R^2^ = 0.350). Trust has a significant impact on repeated purchase intentions (β = 0.431, *p* < 0.01), but the effect of demand uncertainty on repeated purchase intentions is not significant. Finally, the interaction between trust and demand uncertainty was added to the model 3, this leads to model 4. The model 4 fits the data well (R^2^ = 0.351). However, it is found that the coefficient of the interaction term is not significant. Therefore, the uncertainty of demand does not moderate the relationship between trust and repeated purchase intention of agricultural materials, thus Hypothesis 6 is not supported. 

The same method is used to examine the moderation effect of competition intensity. Firstly, model 2 has verified the significant positive impact of trust on repeated purchase intentions. Secondly, the impact of trust and competition intensity on repeated purchase intentions is examined. As shown by model 3, both have significant effects (β = 0.431, *p* < 0.01; β = 0.088, *p* < 0.05). Finally, the interaction term of trust and competition intensity is added to the model 3, this leads to model 5. The fit of model 5 is also good (R^2^ = 0.358). The coefficient of the interaction term is significant (β = −0.601, *p* < 0.05). Therefore, the moderation of the intensity of competition is verified, and the Hypothesis 7 is supported.

To ensure the robustness of the empirical results, the two moderation variables were put in the full model (i.e., model 6) and examined again. The results are completely consistent with the models 4 and 5, indicating the results for the test of the moderation effect of dynamic environment are stable and reliable. Therefore, Hypothesis 6 is not supported, and Hypothesis 7 is supported.

**Figure 2:**
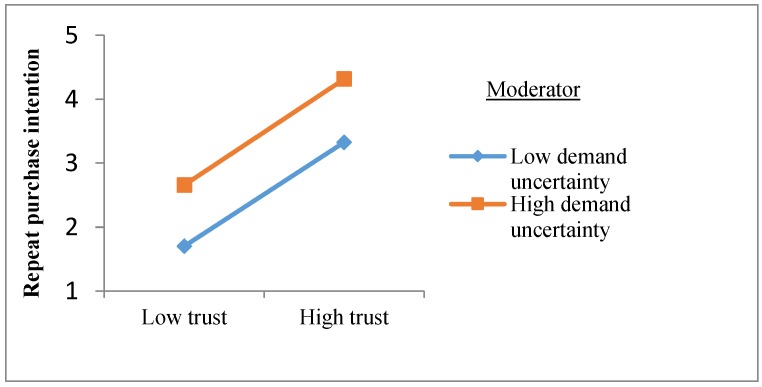
Moderation effect of demand uncertainty on the relationship between trust and repeated purchase intention.

**Figure 3:**
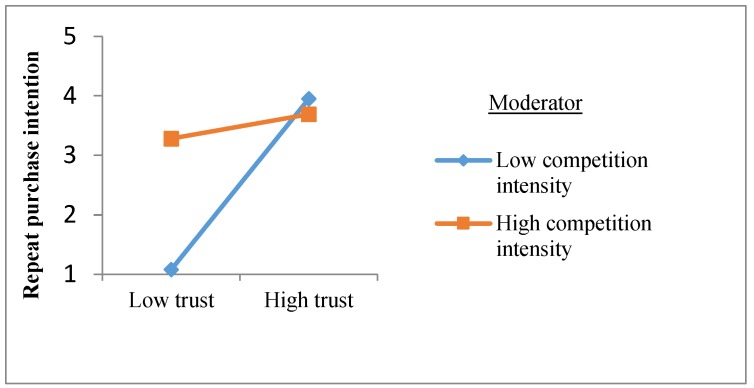
Moderation effect of competition intensity on the relationship between trust and repeated purchase intentions.

In order to more intuitively reveal whether demand uncertainty and competitive intensity have a moderation effect on the relationship between trust and repeated purchase intentions, an interaction diagram of the two variables is shown in Figure 2 and Figure 3. It can be seen from Figure 2 that the high and low demand uncertainty has no significant influence on the relationship between trust and repeated purchase intention, and the moderating effect is not established. As can be seen from Figure 3, the competition intensity negatively moderates the effect of trust on repeat purchase intention. Specifically, in a market environment with high competition intensity, trust has a weaker effect on the repeated purchase intention of agricultural materials; while in a market environment with low competition intensity, there is a strong positive relationship between trust and repeated purchase intentions of agricultural materials. 

### 4.2. Robustness Test

In order to verify that there is no random trend or determined trend in the empirical results, 300 questionnaires were selected by the EXCEL random sampling method from 578 questionnaires for robustness test, and the hierarchical regression analysis was performed again. The results are shown in Table 6.

#### 4.2.1. Comparative Analysis of Models

The R values of the six models vary between 0.008 and 0.046, the values of R^2^ vary from 0.007 to 0.056, and the values of adjusted R^2^ vary between 0.005 and 0.053. Thus, the variance is relatively small and negligible. Therefore, it can be concluded that there is no big difference in the model fitting between 578 questionnaires and 300 questionnaires.

#### 4.2.2. Comparison of the Hypotheses Test Results

Regarding Hypothesis 1, in which the impact of personal relations on trust is to be examined. Both of the regression results of 578 questionnaires in model 1 (β = 0.437, *p* < 0.01) and the regression results of 300 questionnaires (β = 0.441, *p* < 0.01) indicate the relationship is significant and positive, with subtle differences in β values.

Hypothesis 2 proposes the impact of instrumentality on trust. Again, the regression results of 578 questionnaires in model 1 (β = −0.063, *p* < 0.1) and the regression results of 300 questionnaires (β = −0.126, *p* < 0.05) indicate the same effect, i.e., instrumentality is significantly but negatively associated with trust, only that the result for 300 questionnaires were slightly more significant.

Hypothesis 3 proposes that the impact of personal relations on repeated purchase intentions. The regression results of 578 questionnaires in model 2 (β = 0.215, *p* < 0.01) and the regression results of 300 questionnaires (β = 0.239, *p* < 0.01) indicate the same result, i.e., there is a significant positive relation between personal relations and repetitive purchase intentions, only with subtle differences in β values.

Hypothesis 4 proposes the impact of instrumentality on repeated purchase intentions. Both of the regression results of 578 questionnaires in model 2 (β = −0.148, *p* < 0.01) and the regression results of 300 questionnaires (β = −0.210, *p* < 0.01) show the same effect, i.e., instrumentality significantly and negatively associated with repeated purchase intentions, with subtle differences in beta values.

Hypothesis 5 proposes the impact of trust on repeated purchase intentions. Again, the regression results of 578 questionnaires in model 2 (β = 0.443, *p* < 0.01) and the regression results of 300 questionnaires (β = 0.452, *p* < 0.01) shows the same effect that trust is significantly and positively correlated with repeated purchase intentions, with only subtle differences in beta values.

Hypothesis 6 proposes the moderation effect of uncertainty of demand. Neither the results of 578 questionnaires nor the results of 300 questionnaires support the hypothesis. The results were stable and consistent.

Hypothesis 7 proposes the moderation effect of competition intensity on the relationship between trust and repeated purchase intentions. Both of the regression results of 578 questionnaires in model 5 (β = −0.601, *p* < 0.05) and the regression results of 300 questionnaires (β = −0.084, *p* < 0.1) indicate a significant negative moderation effect of competition intensity, except that the significance level of the 578 questionnaires was slightly higher.

In summary, the regression results in Table 5 are consistent with that in Table 4. After extracting about half of the total sample, the relationship between other variables in the model passed the significance test except that the demand uncertainty still did not moderate the relationship between trust and repeated purchase intentions. This confirms that personal relations positively affect trust and repeated purchase intentions; instrumentality negatively and significantly affects trust and repeated purchase intentions; trust positively affects repeated purchase intentions, and the competition intensity negatively moderates the relationship between trust and repeated purchase intentions. It demonstrates that the regression results of the above-mentioned whole sample are robust.

## 5. Discussions

The results of the study show that firstly, the two dimensions of guanxi have different effects on trust. Personal relations play a positive role in generating the trust of farmers in agricultural retailers, while instrumentality plays a negative role in this relationship. Good personal relations mean that the two sides of the transactions have frequent communication and interaction, thus establishing a bridge of mutual trust between them. This result is consistent with the extant research, e.g., Wang [16] and Li [8]. The higher instrumentality means that the transactions between the two parties are short-lived and utilitarian, and the farmers’ perception of the reliability of the agricultural retailers is lower. However, this is inconsistent with the results of Li (2010) in the context of B2B marketing, in which it is argued that instrumentality positively affects interpersonal trust among enterprises. It is analyzed that the salespersons in the B2B situation are professional businessmen, where the most important consideration in dealing with the other party is how much benefits that the other party could bring to them. Therefore, the two parties could predict accurately the other party’s actions based on economic rational. This is also a type of trust, but an ‘instrumental trust’. To obtain expected benefits, e.g., repeated purchase, the seller needs only provide what the buyer wants. Thus, instrumental relationship positively affects instrumental trust between the buyers and sellers in the B2B market. 

While in the agricultural materials market, farmers are not professional businessmen. Their purchasing decisions are very often influenced by factors other than pure economic interests. Especially in Chinese rural areas, due to the information asymmetry and the relatively closed networks, farmers often choose retailers they know well. Thus, making the deal more embedded in emotional elements. In this context, the stronger the instrumentality in the relationship, the less likely trust will be built between the two parties. 

Based on the above discussion, conclusions drawn by previous research, e.g., Peng [15] and Kriz and Fang [14], that guanxi is the basis of trust establishment, should be applied cautiously. This study shows that, in the context of Chinese agricultural materials marketing, only the dimension of personal relations in guanxi is the basis of trust establishment. In this context, when the emotional component is greater than the instrumental component, that is, when the role of personal relations is greater than the instrumentality, the trust between people will be established. 

The results also show that good personal relations lead to the cooperation between farmers and agricultural retailers, thus encourage farmers to choose the same agricultural retailers for new purchases. While the more instrumental the relationship between the two parties is, the more likely they will consider economic interests than other aspects during the business transactions, repeated purchase is thus more unpredictable. In addition, trust lays the foundation for long-term relationships between farmers and agricultural retailers and promotes repeated purchase intentions [38]. Therefore, guanxi between farmers and agricultural retailers could influence repeated purchase intentions of farmers through establishing trust between them. Good personal relations between farmers and agricultural retailers help building trust between the two parties, which in turn leads to more close cooperation between them, and repeated purchase is more likely to happen. While relationship based on pure economic interests does not help trust-building between farmers and agricultural retailers and will unlikely to encourage repeated business transactions between them. 

This is inconsistent with previous research on the relationship between personal relationship and the repeated purchase intention in the B2B and e-commerce market, e.g., Li et al., (2010) [8] and Lin et al., [9], in which while both personal relationship and instrumentality improve the trust between the buyers and sellers, they do not necessarily encourage repeated purchase intention. This is understandable, in those markets, customers are more professional and well informed by product and market information, thus appears more rational and less relied on personal relationship to make repurchase decisions.

Furthermore, demand uncertainty does not have a moderation effect on the relationship between trust and repeat purchase intentions. The reason may be that, due to the relatively fixed planting area, the annual demand for agricultural materials of farmers is basically stable. In addition, farmers have always chosen agricultural materials based on past experience. Therefore, the choice of agricultural brands has not changed much. In this situation, relying on trust to increase farmers’ intention to repeat purchases is more significant than increasing the product variety or adjusting the inventory by agricultural retailers. The intensity of competition moderates the relationship between trust and repeated purchase intentions, which indicates the convenience of transforming agricultural retailers brought about by the intensity of competition negatively affect the role played by trust in the intention of repeated purchases.

Finally, the interaction between guanxi, trust, competition intensity, and repeat purchase intention in the agricultural material market has important implications for food system. Global food security and sustainability is subject to food productivity and the way that food is produced. The application of circularity economy and reuse of food waste to improve the sustainability and security of food supply is gaining a ground in recent years [39,40]. This process is characterized by new agricultural materials to be used, and these might include nutrient inputs produced with emerging technologies for ammonia production, chemical recovery of phosphorus from digested food in sewage, and genetically modified seeds of crops [39]. For Chinese farmers whose knowledge of agricultural material is mainly learned from agricultural retailers, and the utilization of these new materials is largely dependent on the extent to which farmers believe that the retailers’ suggestion would serve their best interests. Therefore, building the trust between farmers and retailers is especially critical to not only the retailers, but also to the circularity and reuse of food waste in food system, and in turn to food security and sustainability. 

Even when some agricultural retailers are not keen to sell materials that are produced from food waste or by new technologies, given that circular economy is increasingly applied to food systems [39,40], intense competition in the market that provides better price, quality, and service would weaken the impact of trust on repeat purchase intention, and attract farmers to switch their purchase to these new retailers. This situation will also promote the security and sustainability of food system.

It is also worth noting that the unique characteristics of Chinese agricultural material market may be changing. The education of the new generation of farmers is improved significantly in recent years. The young generation are better educated and increasingly appear to be more reliant on science and technology to make growing and purchasing decisions. They are more interested in understanding market demands and customer preferences, and more capable of responding to the government’s concern of food security and long-term strategy in developing industrial agriculture [41,42]. Given this situation, it is likely that the role played by personal relationship in Chinese agricultural material market will be weakened, and the importance of instrumentality will be increased. This tendency deserves research attention in the future, and it will be interesting to find out if, and to what extent, the changes in young generations of Chinese farmers will affect the interaction between guanxi, trust, and repeated purchase intention in Chinese agricultural material market.

### 5.1. Theoretical Contributions

This study makes several important theoretical contributions. Firstly, it extends the theory of relationship marketing by showing that the impact of guanxi on the trust varies crossing different contexts. The empirical examination of this study proves that instrumentality has a negative impact on trust in the context of agricultural material marketing in China, while the extant research shows the contrary in the B2B marketing. Secondly, this study enriches scholarly understanding of the impact of guanxi on repeated purchase intentions by showing that the former may have a different effect on the latter in different marketing contexts. Previous studies have confirmed the positive role played by guanxi in repetitive purchase intentions [8,9,11,38], the result of this study shows that the effect might not be positive in the context of Chinese agricultural marketing. Again, as a dimension of guanxi, instrumentality affects repeated purchase intentions of Chinese farmers negatively. This conclusion is obviously different from the extant research, indicating that the marketing context plays an important role in the effect of guanxi on repeated purchase intentions. 

Thirdly, this study extends scholarly understanding of the effect of guanxi on repeated purchase intentions by showing that this effect may be transferred through trust, i.e., trust can be an influencing mechanism between the two. The integration of the three variables—i.e., guanxi, trust, and repeated purchase intentions—in the conceptual model shows that the interaction between them in a special context, such as Chinese agricultural material marketing, could effectively explain the repeated purchase decisions of buyers. In a special context of Chinese rural area, where Chinese traditional culture plays an important role in the social life, relationship marketing practice may well be different from that is in other contexts.

Finally, this study enriches the scholarly understanding of repeated purchase intentions by showing that the effects of some important antecedents, such as guanxi and trust, could be influenced by environmental conditions, such as competition intensity in the marketplace. Different from the previous literature of agricultural marketing, in which the external marketing and consumers’ own characteristics are the focus of discussion, this study examines the relationship between trust and repeated purchase intentions based on the dynamic environment. The research results show that the more intense the competition between agricultural retailers is, the less the role of trust can play, and the dynamic environment will affect the effect of trust on repeated purchase intentions. 

### 5.2. Implications for Management

The results of this study have important implication for practice. Firstly, it shows that the role of personal relations played in agricultural materials market is significant. This suggests that agricultural retailers should properly manage guanxi with farmers, paying particular attention to the establishment and maintenance of personal relations with them, and weaken farmers’ instrumental choices, thus increasing their repeated purchase intentions.

Secondly, trust plays a key role in the purchase of agricultural materials. This suggests that agricultural retailers should emphasize cultivating farmers’ trust in themselves. Agricultural retailers ought to take actions such as improving the quality of agricultural materials, keeping promises to farmers, providing satisfactory after-sales services, and upgrading their professional skills. So that trust can be built and repeated purchase intentions can be obtained subsequently. 

Finally, the highly competitive environment has a negative impact on the economic interests of agricultural retailers. This suggests that agricultural retailers should aware the competition intensity in their industries and adopt appropriate marketing strategies. In the case of high competition, agricultural retailers ought to take extra efforts, such as offering preferential prices, credit sales services and door-to-door delivery to maintain customers. Therefore, farmers received more ‘real’ benefits, and perceive the deals as ‘big bargains’. On the contrary, if the competition in the market is relatively weak, agricultural retailers should pay more attention to cultivating farmers’ trust to maintain customers and achieve more sales.

### 5.3. Limitations and Further Research

Although the study has important contributions to the literature, it is not without limitations. Firstly, the agricultural materials, the purchase of agricultural materials and the rural social network in Chinese rural area compose a special context of this study. Therefore, caution is advised when applying the results of this research to other contexts.

Secondly, this study focuses on the two dimensions of guanxi—i.e., personal relations and instrumentality—other dimensions such as human feelings and face have not been considered. Therefore, more elements of guanxi should be explored in the future, thus a deeper and more comprehensive understanding of the effect of guanxi on repeated purchase intentions could be achieved.

Thirdly, the data collected only from four provinces of the eastern, central, and western regions of China, but did not reach a wider region, thus the generalization of the research results needs further investigation. In addition, this paper uses only horizontal data but not longitude data, it is more conducive to discover the change of data along time span. Therefore, longitudinal research design can be considered in the future to verify the causal relationship in the conceptual model.

Finally, as discussed above, the Chinese agricultural material market is changing, especially the education of young generation of farmers is improved significantly in recent years, combined with new technology, and the development of industrial agriculture, and the long-term strategy of government in food security, the interaction between guanxi, trust, and repeated purchase intention in Chinese agricultural material market is very likely to change. Thus, a dynamic perspective must be applied to understand the conclusions of this study. At the same time, it will be interesting to conduct a research in a few years time to find out if and to what extent the expected change would happen. 

## 6. Conclusions 

This study explores the influencing factors of repeated purchase intentions of farmers in the agricultural resource market in China. In particular, it examines the impact of guanxi and trust on repeated purchase intention, and the moderating effect of dynamic environment on the relationship between trust and repeated purchase intentions. A data set of 578 samples was used and hierarchical regression analysis was conducted to examine the conceptual models. The results generally support the hypotheses that based on the conceptual model. The results show that guanxi between farmers and agricultural retailers has a positive effect on trust between them and on repeated purchase intentions of farmers. While instrumentality has a negative effect on trust between them and on repeated purchase intentions of farmers. The trust between farmers and agricultural retailers promotes farmers’ repeated purchase intentions. The intensity of competition negatively moderates the positive relation between trust and repeated purchases. Demand uncertainty does not moderate the positive effect of trust on repeated purchases. The results and discussion shed light on the agricultural food system sustainability from a dynamic environment embedded business relationship perspective. They also suggest that conclusions drawn by previous research that based on B2B and e-commerce market may not be applicable to Chinese agricultural material market, where customers’ background and their interaction with suppliers are of unique characteristics. 

## Figures and Tables

**Table 1:** Sample profile (%)

Variables	Profile
**Gender**	
Male	66.78
Female	33.22
**Farming Experience**	
≤10 years	23.9
11–30 years	38
≥31 years	38.1
**Education**	
Less literacy	10.38
Elementary school	31.66
Junior high school	45.67
High school/technical school	11.07
Diploma and above	1.21
**Agri. Income/Total Income**	
≤25%	50.9
26–50%	27.8
≥51%	21.3
**Age**	
≤36	10.38
37–46	26.12
47–54	27.85
55–65	23.70
**Family Income (Yuan)**	
≤10,000	25.78
10,000–30,000	47.58
30,000–60,000	19.55
60,000–90,000	3.80
>90,000	3.29

**Table 2:** Reliability and validity

Scale	Factor Loadings
**Personal relations (CR = 0.837, AVE = 0.507, α = 0.752, Percentage of variance explained = 50.74%)**	
When local farmers interact with sales staff in agricultural retail stores, they think each other is a friend of their own.	0.725
Local farmers are willing to help each other on non-work issues when they interact with sales staff at agricultural retail stores.	0.712
Local farmers often talk about some personal issues when they interact with salespeople in agricultural retail stores.	0.627
When local farmers interact with the sales staff of the agricultural retail store, even if the current buying and selling relationship is over, they will keep in constant contact with each other.	0.736
Local farmers think of each other as a circle when they interact with sales staff at agricultural retail stores.	0.688
**Instrumentality (CR = 0.919, AVE = 0.850, α = 0.825, Percentage of variance explained = 85.08%)**	
If local farmers are not buying agricultural materials (pesticide, fertilizer, seeds), they are not willing to contact agricultural retail stores.	0.922
I believe that if there is not a demand for agricultural materials, local farmers will not be willing to contact agricultural retail stores.	0.922
**Trust (CR = 0.876, AVE = 0.542, α = 0.826, Percentage of variance explained = 54.16%)**	
Agricultural retail stores are committed to us.	0.657
We believe that the information provided by agricultural retail stores.	0.775
Agricultural retail stores really care about our agricultural harvest.	0.753
When making important decisions, the agricultural retail store will consider giving both-sides benefits.	0.764
We believe that agricultural retail stores are always concerned about our interests.	0.696
Agricultural retail stores are worthy of trust.	0.764
**Uncertainty in demand (CR = 0.870, AVE = 0.695, α = 0.775, Percentage of variance explained = 69.45%)**	
The demand of local farmers in the agricultural retail industry is difficult to predict.	0.754
Local farmers in the agricultural retail industry always seek new differences.	0.864
The preferences of local farmers in the agricultural retail industry are always changing.	0.877
**Competition intensity (CR = 0.793, AVE = 0.560, α = 0.607, Percentage of variance explained = 56.07%)**	
There are many agricultural retail stores in the agricultural retail market that provide similar agricultural materials (pesticide, fertilizer, seeds).	0.704
Agricultural materials (pesticide, fertilizer, seeds) in the agricultural retail industry are changing rapidly.	0.677
The market competition of agricultural materials retail industry is very fierce.	0.752
**Repeat purchase intention (CR = 0.878, AVE = 0.707, α = 0.789, Percentage of variance explained = 70.70%)**	
Local farmers will have more business dealings with agricultural retail stores in the future.	0.763
Local farmers will purchase new agricultural materials (pesticide, fertilizer, seeds) or new services provided by frequent agricultural retail stores.	0.881
Local farmers will buy more agricultural materials (pesticide, fertilizer, seeds) or services from frequent agricultural retail stores.	0.873

Note. CR = construct reliability; AVE = Average Variance Extracted.

**Table 3:** KMO and Bartlett sphericity test.

KMO and Bartlett Sphericity Test		Personal Relations	Instrumentality	Trust	Demand Uncertainty	Competition Intensity	Repeated Purchase Intention
Kaiser–Meyer–Olkin		0.756	0.500	0.830	0.664	0.637	0.669
Bartlett sphericity test	Approximate chi-square distribution	663.836	390.141	1189.210	530.125	183.003	571.656
	Freedom	10	1	15	3	3	3
	Significant probability	0.000	0.000	0.000	0.000	0.000	0.000

**Table 4:** Correlation coefficient between statistical description and each research variable.

Variables	Means	Standard Deviation	1	2	3	4	5	6
1. Repeat purchase intention	5.641	0.996	(0.841)					
2. Personal relations	5.012	1.088	0.392 **	(0.712)				
3. Instrumentality	4.001	1.665	−0.137 **	0.095	(0.922)			
4. Trust	5.352	0.960	0.539 **	0.431 **	−0.022	(0.736)		
5. Demand uncertainty	4.737	1.372	0.105 *	0.236 **	0.160 **	0.144 **	(0.834)	
6. Competition intensity	5.555	0.986	0.211 **	0.197 **	0.017	0.199 **	0.228 **	(0.748)

Note: The number of samples is *N* = 578; the value in parentheses on the diagonal is the square root of the mean variation extraction (AVE). The non-diagonal is the correlation coefficient of each variable, ** *p* < 0.01 means significant at 99% confidence, and * *p* < 0.05 means significant at 95% confidence.

**Table 5:** Results of hierarchical regression.

Variables	Trust (TR)	Repeat Purchase Intention (RI)				
	Model 1	Model 2	Model 3	Model 4	Model 5	Model 6
Personal relation (PR)	0.437 ***	0.215 ***	0.203 ***	0.203 ***	0.205 ***	0.204 ***
Instrumentality (IV)	−0.063 *	−0.148 ***	−0.148 ***	−0.149 ***	−0.153 ***	−0.153 ***
Trust (TR)		0.443 ***	0.431 ***	0.430 ***	0.810 ***	0.820 ***
Uncertainty in demand (DU)			−0.001	0.002	0.003	0.002
Competition intensity (CI)			0.088 **	0.088 **	0.475 ***	0.486 ***
Uncertainty in demand × Trust (DU × TR)				−0.017		0.008
Competition intensity × Trust (CI × TR)					−0.601 **	−0.616 **
R	0.436	0.586	0.592	0.592	0.598	0.598
R^2^	0.190	0.343	0.350	0.351	0.358	0.358
ΔR^2^	0.187	0.340	0.345	0.344	0.351	0.350
F	67.465	99.950	61.734	51.418	52.960	45.325

Note: ***, *p* < 0.01; **, *p* < 0.05; *, *p* < 0.1.

**Table 6:** Results of hierarchical regression analysis (after selecting 300 samples)

Variables	Trust (TR)	Repeated Purchase Intention (RI)				
	Model 1	Model 2	Model 3	Model 4	Model 5	Model 6
Personal relations (PR)	0.441 ***	0.239 ***	0.236 ***	0.235 ***	0.245 ***	0.244 ***
Instrumentality (IV)	−0.126 **	−0.210 ***	−0.207 ***	−0.205 ***	−0.213 ***	−0.210 ***
Trust (TR)		0.452 ***	0.447 ***	0.449 ***	−0.021 ***	0.433 ***
Uncertainty in demand (DU)			−0.026	−0.033	0.068	−0.031
Competition intensity (CI)			0.057	0.055	0.433	0.066
Uncertainty in demand × Trust (DU × TR)				0.038		0.059
Competition intensity × Trust (CI × TR)					−0.084 *	−0.096 **
R	0.444	0.632	0.634	0.635	0.639	0.642
R^2^	0.197	0.399	0.402	0.403	0.409	0.412
ΔR^2^	0.192	0.393	0.392	0.391	0.397	0.398
F	36.530	65.474	39.529	33.022	33.756	29.210

Note: ***, *p* < 0.01; **, *p* < 0.05; *, *p* < 0.1.

## Appendix A

**Appendix Questionnaire**

### Completion Instructions:

Dear Sir/Madam:

Thank you very much for taking the time to participate in the research activities carried out by the research group of “Trust Transfer Mode and Marketing Strategy Research in Agricultural Sales”.

In order to obtain accurate data, please answer or fill in the relevant questions according to the actual situation. We will treat all information you provide in absolute confidence. Scientific research, policy analysis, and opinion review are published in a large number of questionnaires, rather than the case information of your individual, family, and village, and will not cause leakage of your personal, family, and village information. We apologize for any inconvenience caused to your life and agricultural production, and thank you for your understanding and assistance in our academic research.

Please fill in the relevant information or mark “√” on the number you think is appropriate. Sincerely thank you for your support and help in the development of China’s business management discipline.

### Basic Information:

01.The type of respondent: 1) ordinary farmers; 2) farming expert; 3) village cadres; 4) agricultural technicians
02. Address of the village: _________ City __________ County _________ Township _______ VillageYour name: _____________ Contact number: ____________
03. Your gender: 0—male; 1—female
04. Your age: 1) under 36 years old; 2) 37~46 years old; 3) 47~54 years old; 4) 55~65 years old; 5) over 66 years old
05. Education level: 1) less literacy; 2) elementary school; 3) junior high school; 4) high school or technical school; 5) college and above
06. Farming experience: 1) 10 years and below; 2) 11 to 30 years; 3) 30 years or more
07. Size of your farm: 1) 5 mu and below; 2) 6 ~ 15 mu; 3) more than 15 mu
08. Type of land: 1) cultivated land; 2) mountainous land; 3) forest fruit land; 4) water surface; 5) pasture
10. The average annual income of your family: 1) less than 10,000 (inclusive) yuan; 2) 10,000 to 30,000 (inclusive) yuan; 3) 40,000 to 60,000 (inclusive) yuan; 4) 70,000 to 90,000 (inclusive) yuan; 5) 100,000 yuan or more
11. Agricultural income as a percentage of total income: 1) 25% and below; 2) 26% ~ 50%; 3) 51% or more

Please choose according to the actual situation: 1—completely disagree; 2—basically disagree; 3—do not agree; 4—does not matter; 5—partially agree; 6—basically agree; 7—fully agree								
**Guanxi**								
**Personal relations**								
S1.1	When local farmers interact with sales staff in agricultural retail stores, they think each other is a friend of their own.	1	2	3	4	5	6	7
S1.2	Local farmers are willing to help each other on non-work issues when they interact with sales staff at agricultural retail stores.	1	2	3	4	5	6	7
S1.3	Local farmers often talk about some personal issues when they interact with salespeople in agricultural retail stores.	1	2	3	4	5	6	7
S1.4	When local farmers interact with the sales staff of the agricultural retail store, even if the current buying and selling relationship is over, they will keep in constant contact with each other.	1	2	3	4	5	6	7
S1.5	Local farmers think of each other as a circle when they interact with sales staff at agricultural retail stores.	1	2	3	4	5	6	7
S1.6	When the local farmers interact with the sales staff of the agricultural retail store, the relationship between the two parties is tested for a long time.	1	2	3	4	5	6	7
**Instrumentality**								
S2.1	Local farmers maintain their relationship with agricultural retail stores and contribute to the choice of agricultural products (pesticide, fertilizer, seeds)	1	2	3	4	5	6	7
S2.2	Local farmers maintain relationships with agricultural retail stores and help to obtain information or resources on agricultural products (pesticide, fertilizer, seeds)	1	2	3	4	5	6	7
S2.3	Local farmers maintain their relationship with agricultural retail stores, helping to reduce the cost of agricultural products (pesticide, fertilizer, seeds)	1	2	3	4	5	6	7
S2.4	If local farmers are not buying agricultural materials (pesticide, fertilizer, seeds), they are not willing to contact agricultural retail stores.	1	2	3	4	5	6	7
S2.5	I believe that if there is not a demand for agricultural materials, local farmers will not be willing to contact agricultural retail stores.	1	2	3	4	5	6	7
**Dynamic environment**								
**Demand uncertainty**								
T1.1	The demand of local farmers in the agricultural retail industry is difficult to predict.	1	2	3	4	5	6	7
T1.2	Local farmers in the agricultural retail industry always seek new differences.	1	2	3	4	5	6	7
T1.3	The preferences of local farmers in the agricultural retail industry are always changing.	1	2	3	4	5	6	7
**Competition intensity**								
T2.1	There are many agricultural retail stores in the agricultural retail market that provide similar agricultural materials (pesticide, fertilizer, seeds).	1	2	3	4	5	6	7
T2.2	Agricultural materials (pesticide, fertilizer, seeds) in the agricultural retail industry are changing rapidly.	1	2	3	4	5	6	7
T2.3	In the agricultural retail industry, the strategy of price competition is often adopted between agricultural retail stores.	1	2	3	4	5	6	7
T2.4	The market competition of the agricultural materials retail industry is very fierce.	1	2	3	4	5	6	7
**Trust**								
X2.1	Agricultural retail stores are committed to us.	1	2	3	4	5	6	7
X2.2	Agricultural retail stores do not always treat us honestly (R).	1	2	3	4	5	6	7
X2.3	We believe the information provided by agricultural retail stores.	1	2	3	4	5	6	7
X2.4	Agricultural retail stores really care about our agricultural harvest.	1	2	3	4	5	6	7
X2.5	When making important decisions, the agricultural retail store will consider giving both-sides benefits.	1	2	3	4	5	6	7
X2.6	We believe that agricultural retail stores are always concerned about our interests.	1	2	3	4	5	6	7
X2.7	Agricultural retail stores are worthy of trust.	1	2	3	4	5	6	7
X2.8	We found it necessary to be cautious about agricultural retail stores (R).	1	2	3	4	5	6	7
**Repeat purchase intention**								
Z2.1	Local farmers will have more business dealings with agricultural retail stores in the future.	1	2	3	4	5	6	7
Z2.2	Local farmers will purchase new agricultural materials (pesticide, fertilizer, seeds) or new services provided by frequent agricultural retail stores.	1	2	3	4	5	6	7
Z2.3	Local farmers will buy more agricultural materials (pesticide, fertilizer, seeds) or services from frequent agricultural retail stores.	1	2	3	4	5	6	7
